# Co-infection with *Legionella* and SARS-CoV-2: a case report

**DOI:** 10.1186/s40981-021-00467-3

**Published:** 2021-08-18

**Authors:** Masaru Shimizu, Yusuke Chihara, Sakiko Satake, Astuko Yone, Mari Makio, Hideki Kitou, Tomohiro Takeda

**Affiliations:** 1 Department of Anesthesia and Perioperative Care, University of California San Francisco, 505 Parnassus Ave, San Francisco, CA 94143 USA; 2 Department of Pulmonary Medicine, Uji-Tokushukai Medical, 145 Ishibashi Makishimacho, Uji, Kyoto Japan; 3 Department of Anesthesiology, Uji-Tokushukai Medical, 145 Ishibashi Makishimacho, Uji, Kyoto Japan

**Keywords:** *Legionella*, SARS-CoV-2, Co-infection, Pneumonia, Early diagnosis, Early treatment

## Abstract

**Introduction:**

We report a case of COVID-19 with *Legionella* co-infection that was treated successfully.

**Case report:**

A 73-year-old man presented to the hospital with symptoms of fatigue that continued for the next 5 days. The patient was receiving docetaxel and prednisolone chemotherapy for prostate cancer. Laboratory findings on admission showed positive urine *Legionella* antigen test and SARS-CoV-2 test. He was administered antiviral and antibacterial agents, and a corticosteroid. Pneumonia exacerbated on day 2 of hospitalization. The patient underwent tracheal intubation and began receiving multidisciplinary care. On day 8 of hospitalization, his oxygenation improved, and the patient was extubated. He discharged on day 27 of hospitalization.

**Conclusions:**

The patient had a favorable outcome with early diagnosis and early treatment of both diseases. Patients with severe COVID-19 disease need to be evaluated for co-infection. Further, early diagnosis and early treatment of the microbial bacteria causing the co-infection are important.

## Introduction

Severe acute respiratory syndrome coronavirus-2 (SARS-CoV-2) is a novel coronavirus that causes coronavirus disease 2019 (COVID-19). As of 24 October 2020, the WHO reports that there are 41.8 million patients diagnosed with COVID-19 and 1.13 million deaths worldwide. COVID-19 patients present primarily with fever and respiratory tract symptoms. However, these symptoms also occur in respiratory infections caused by other microorganisms. Current reports of co-infection between COVID-19 and respiratory pathogens are increasing worldwide [1]. Fifty percent of patients who died of COVID-19 had a secondary bacterial infection [2]. We present a case of co-infection involving *Legionella* and SARS-CoV-2, in which early diagnosis and early treatment were useful.

## Case report

**Fig. 1:**
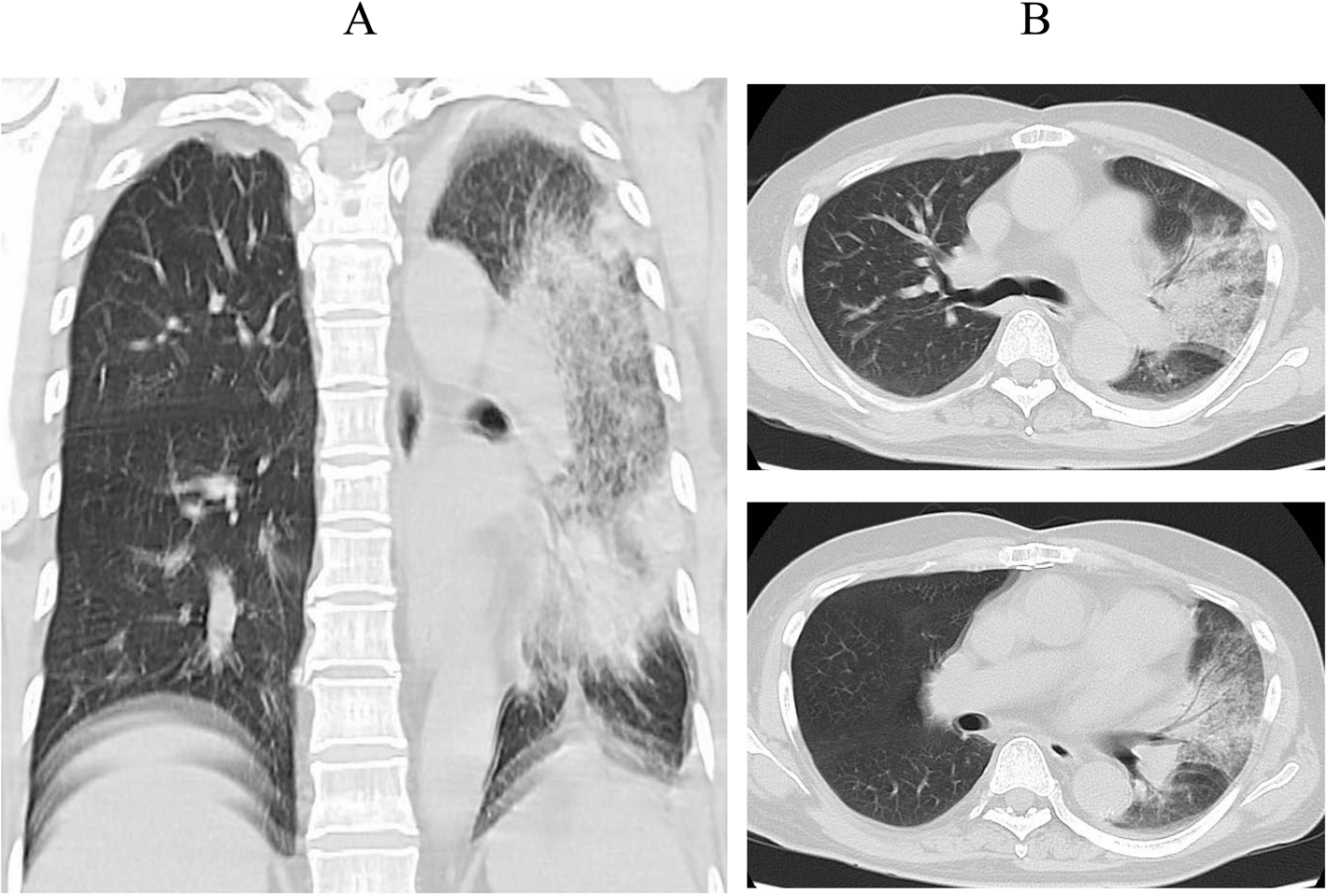
Chest computed tomography (CT) on admission. Chest CT on contrast coronal (**A**) and axial (**B**) views showing infiltrative shadow and pleural effusion in the upper and lower left lobes

The patient was a 73-year-old man. He received docetaxel and prednisolone chemotherapy for prostate cancer and was administered pegfilgrastim after chemotherapy. His medication history included oral administration of rivaroxaban. Eight days before the onset of fatigue, he was traveling during chemotherapy and had been in a hot tub. Subsequently, he visited the hospital because he continued to experience fatigue over the next 5 days. The patient's physical findings were as follows: alert and oriented, fever (39.0 °C), dyspnea (oxygen saturation [SpO_2_] 90% with no oxygen; respiratory rate, 17 breaths/min), no hemoptysis, and no chest pain. The patient’s blood pressure was normal (111/81 mmHg); however, tachycardia and atrial fibrillation (132 beats/min) were present. The A-DROP score for assessing *the severity of community-acquired pneumonia* by the Japanese Respiratory Society was 3 points (age ≥ 70 years in men, dehydration, or blood urea nitrogen ≥ 21 mg/dL, SpO2 ≤ 90%). The patient was admitted to the hospital for severe pneumonia. On admission, the patient’s white blood cell count was 700/μL; serum sodium, 133 mEq/L; C-reactive protein (CRP), 28 mg/dL; serum lactate dehydrogenase (LDH) concentration, 335 U/L; urea nitrogen, 22.4 mg/dL; serum creatinine, 1.23 mg/dL; and D-dimer level, 1.8 μg/mL. Chest CT revealed an infiltrative shadow and pleural effusion in the upper and lower left lobes (Fig. 1) but did not show the appearance of a typical COVID-19 radiological pattern including ground-glass opacities (GGOs) in both inferior lobes.

**Fig. 2:**
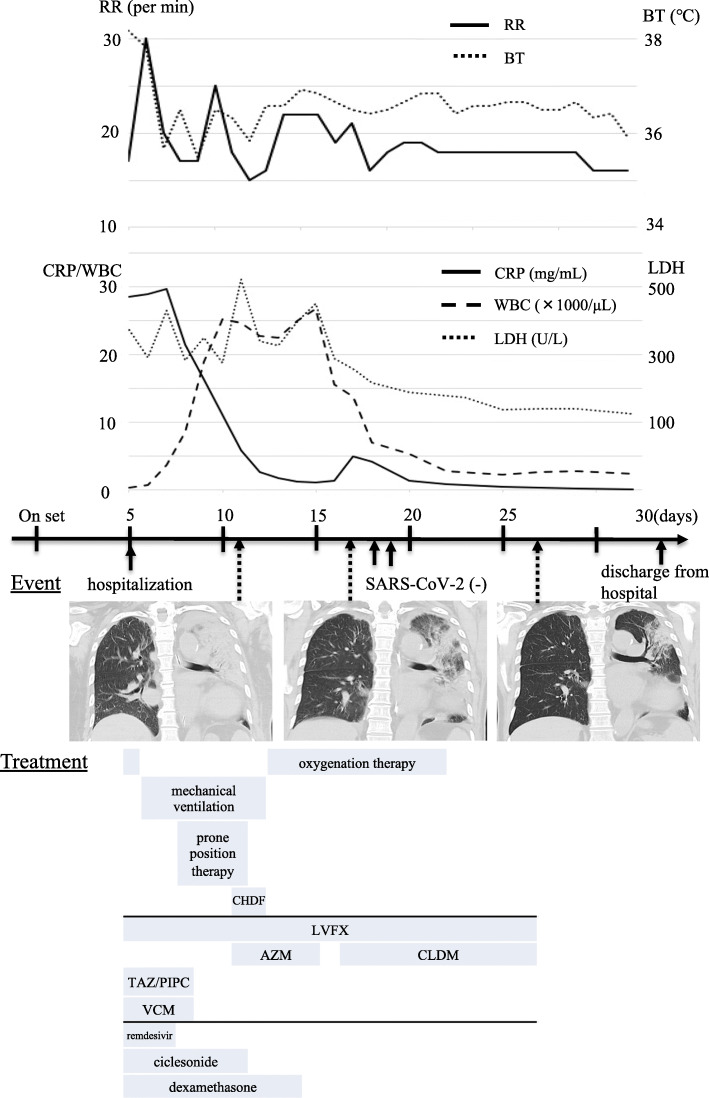
Clinical course and changes in laboratory and computed tomography (CT) findings and vital signs. A time series of the clinical course including relevant laboratory and CT findings and vital signs is shown. RR, respiratory rate; BT, blood temperature; CRP, C-reactive protein; WBC, white blood cell; LDH, lactate dehydrogenase; SARS-CoV-2: severe acute respiratory syndrome coronavirus-2; LVFX, levofloxacin; AZM, azithromycin; CLDM, clindamycin; CHDF, continuous hemodiafiltration; TAZ/PIPC, tazobactam/piperacillin; VCM, vancomycin

The urine *Legionella* antigen test by immunochromatography and SARS-CoV-2 test by loop-mediated isothermal amplification (LAMP) of swabs from the nasal cavity were positive. The patient was immediately administered levofloxacin, tazobactam/piperacillin, vancomycin, remdesivir, ciclesonide, nafamostat mesilate, and dexamethasone. On day 2 of hospitalization, the patient’s dyspnea worsened, his respiratory rate increased to 30 breaths/min, and chest radiograph revealed worsening pneumonia. Therefore, tracheal intubation was performed. The partial pressure of oxygen/fraction of inspired oxygen ratio (P/F ratio) after intubation was 160, indicating acute respiratory distress syndrome (ARDS). Subsequently, the patient was provided with analgesia, sedation, and hemodynamic maintenance. After tracheal intubation, respiratory management, including a tidal volume of ≤ 6 mL/kg/ideal body weight, control of plateau pressure, and high positive end-expiratory pressure were provided according to the lung protection strategy of ARDS. One day after intubation, his P/F ratio improved to 390, but 2 days after intubation, his P/F ratio worsened to 220, so prone position therapy was started. On day 6 of hospitalization, his serum creatinine increased to 3.22 mg/dL, and continuous hemodiafiltration was started. On day 8 of hospitalization, his P/F ratio improved to 361, and he was extubated. His general condition also improved, and his serum creatinine decreased to 2.3 mg/dL. The electrolyte imbalance was not recognized, so the continuous hemodiafiltration was discontinued. He was moved to the general ward on the 14th day of hospitalization. On the day 20, oxygen administration was no longer necessary, and the patient was discharged on the 27th day of hospitalization (Fig. 2).

## Discussion

Here, we present a successful case of early diagnosis and treatment of a patient with severe pneumonia and co-infection with *Legionella* and SARS-CoV-2. The following two conclusions were drawn from this case: first, COVID-19 patients should be examined keeping in mind the possibility of co-infection. Second, early diagnosis and treatment of the bacteria causing the co-infection are important.

COVID-19 patients should be examined with the possibility of co-infection in mind. Seven percent of all COVID-19 patients admitted were co-infected with bacteria. Fourteen percent of patients with severe COVID-19 disease admitted to the ICU are co-infected, with *Mycoplasma* being the most common bacterium causing co-infections, followed by *Pseudomonas aeruginosa*. Among viruses, respiratory syncytial virus (RSV) is the most common, followed by influenza A [3]. Co-infection with *Legionella* and SARS-CoV-2 is extremely rare, and only a few cases have been reported [4, 5]. *Legionella* is an important causative agent of community-acquired and nosocomial pneumonia, accounting for 3–8% of all pneumonia cases [6, 7]. Host predisposing factors to *Legionella* pneumonia include male sex, smoking, chronic lung disease, immunocompromised states, and extremes of age. Environmental predisposing factors include exposure to contaminated water supplies, use of public transportation, and overnight travel. In this case, we suspected *Legionella* pneumonia because the patient had some risk factors and radiological pattern, including GGOs in both inferior lobes, was not typical of COVID-19 [8]. The patient had no known close contact with COVID-19 patients, but we strongly suspected pneumonia because of fever and respiratory symptoms, so we also conducted a SARS-CoV-2 LAMP diagnostic assay. However, given the unprecedented circumstances of COVID-19 and the burden on hospitals, patients may not have undergone thorough microbiological testing [9]. It is not possible to diagnose co-infection only by blood tests, imaging tests, and clinical findings. Therefore, co-infection in COVID-19 patients may be overlooked and underestimated without being diagnosed.

The mechanisms of co-infection of SARS-CoV-2 with *Legionella* and other microbial bacteria are unknown. Necropsy of patients who had SARS-CoV-2 pneumonia, which is similar to influenza virus pneumonia, revealed lymphocytic inflammation with diffuse alveolar damage, chronic inflammation, and bronchial mucosal edema [10–14]. These studies demonstrate that SARS-CoV-2 pneumonia causes damage to the tissues of the bronchi and alveolar epithelium, creating a favorable environment for bacterial growth and adhesion, and promoting invasion and severe inflammation [15]. Co-infection by bacteria during a viral infection increases mortality, and risk factors include age and immunosuppression [16, 17]. Risk factors for aggravation of the disease in patients with *Legionella* pneumonia were chronic obstructive pulmonary disease, smoking, age 50 years and older, male sex, malignancies, immunodeficiency, and renal impairment [8, 18]. Risk factors for rapid progression of symptoms in COVID-19 patients were age and immunosuppression [19–21]. In other words, when treating elderly patients with immunosuppression, we must consider co-infection. The patient in this case had pancytopenia caused by anticancer therapy and was immunosuppressed. Furthermore, early diagnosis and early treatment of the microbial bacteria that cause co-infection are vital. The family *Legionellaceae* has more than 60 species and more than 80 serogroups. *Legionella pneumophila* (*L. pneumophila*) is the most common species, and it causes 90% of the cases of legionellosis [6, 7]. *L. pneumophila* was detected in this case using the immunochromatographic urinary *Legionella* antigen test, Ribotest Legionella (Asahi Kasei Pharma Co., Tokyo, Japan). This test can detect all serotypes of *L. pneumophila*. Therefore, early diagnosis was possible in this case.

Conventional microbiological tests are usually performed for all species of *Legionellaceae*, except *L. pneumophila*; however, smearing, culturing, identification, and drug susceptibility testing take approximately 3 days to obtain results. Patients infected with *Legionella* pneumonia are difficult to distinguish from those infected with other bacterial pneumonia, such as *Streptococcus pneumoniae*, based on clinical symptoms and physical findings. Beta-lactams, the first-line drugs for treating bacterial pneumonia, are ineffective against *Legionella* pneumonia. Therefore, it is necessary to administer macrolides and novel quinolones that are effective against intracellularly parasitic *Legionella*. Patients with legionellosis become severely ill due to respiratory failure, causing rapid exacerbation of symptoms. The mortality rate of patients admitted to the intensive care unit is 25–40% [22, 23]. Therefore, clinicians must determine the causative microorganism based on patient interview, clinical symptoms, blood test results, imaging findings, patient background, and infection site, and promptly begin the treatment**.** Although the rate of bacterial co-infection in COVID-19 patients was low, 70% of patients received antibiotics [3]. However, there is insufficient evidence to support the widespread use of empirical antibiotics in non-severe COVID-19 patients. Patients with respiratory infections are currently experiencing problems such as improper medication administration, unnecessary tests, and inefficient hospital bed management. Therefore, treatment of infectious diseases, in which the course of the disease depends on the treatment strategy in the acute phase, requires faster and more accurate information. Blood test findings for an early presumptive diagnosis of *Legionella* pneumonia were decreased serum sodium and platelets, increased CRP, and increased serum LDH levels [24–26]. Chest findings for *Legionella* pneumonia were non-specific [8]. Blood test findings useful for diagnosing COVID-19 included lymphocyte depletion, elevated LDH concentration, and increased D-dimer [27]. Early CRP levels in critical COVID-19 patients averaged 10.5 mg/dL [28]. Early chest CT findings in COVID-19 patients showed bilateral ground glass opacities of more than 87%, but little pleural effusion [29]. In this case, CRP was 28 mg/dL, which was higher than the mean CRP in severe COVID-19 cases, and imaging findings revealed infiltrative shadow and pleural effusion. Therefore, the possibility of co-infection was considered likely.

Next, a reliable diagnosis is required. Therefore, point-of-care testing (POCT) involving a simple, quick, and accurate test is important. The benefits of POCT are most apparent when location, environment, personnel, and infrastructure are severely constrained. POCT mainly includes antigen and genetic testing. The urinary antigen test used for diagnosing legionellosis in this case is the fastest and is widely recognized for its usefulness. In recent years, genetic testing has improved in terms of technology, automation, cost, and rapidly expanding targets. For respiratory, intestinal, and bloodstream infections, approximately 15 to 20 types of pathogens, including viruses and bacteria, can be detected simultaneously [30–32]. The introduction of rapid genetic testing will improve the quality of diagnosis and treatment of individual cases. This will impact not only individual medical care policies, but also the overall proper use of antibacterial agents [33], infection control [34, 35], and total medical expenses [35, 36].

## Conclusion

Patients admitted with COVID-19 should be thoroughly evaluated for a possible co-infection with other respiratory microorganisms. POCT, a simple, rapid, and accurate test, should be performed for COVID-19 patients suspected of having a co-infection based on clinical findings and for those who are likely to become severely infected. It is desirable to make a definitive diagnosis at an early stage and to begin early treatment, thereby preventing both treatment and antibacterial drug abuse. This approach has several advantages including suppression of resistant bacterial growth and reduction of medical expenses.

## Data Availability

All data generated or analyzed during this study are included in this published article.

## Abbreviations

ARDS: Acute respiratory distress syndrome
COVID-19: Coronavirus disease 2019
CRP: C-reactive protein
GGOs: Ground-glass opacities
*L. pneumophila*: *Legionella pneumophila*
LAMP: Loop-mediated isothermal amplification
LDH: Lactate dehydrogenase
P/F ratio: Partial pressure of oxygen/fraction of inspired oxygen ratio
POCT: Point-of-care testing
RSV: Respiratory syncytial virus
SARS-CoV-2: Severe acute respiratory syndrome coronavirus-2
SpO_2_: Peripheral oxygen saturation
